# Care of the Perineum during and after Labor*Read before the Brazos Valley Medical Association, November 13, 1901, at Bryan, Texas.

**Published:** 1902-07

**Authors:** E. O. Boggs

**Affiliations:** Marquez, Texas


For Texas Medical Journal.

### Care of the Perineum During and After Labor.*

BY E. O. BOGGS, M. D., MARQUEZ, TEXAS.

I shall make my remarks very brief, and give my ideas and methods as I have carried them out in practice. The first care of the accoucheur should of course be directed to preparing the perineum, for the great strain that will be exerted upon it in the passage of the head, or breech, as the case may be.

In a practice extending over a period of eight years, I have been fortunate in having but three (3) cases of lacerated perineum extensive enough to require repairing. Of the three, two were cases of instrumental delivery, and in the third, the rigid os and the unyielding perineum was attributed to the age of the mother, some thirty-five or thirty-six years. All three being primipara.

Lusk says: "The condition termed rigidity of the perineum is usually the sign of failing uterine action," yet in the foregoing case last mentioned, at the expiration of six hours of hard or strong labor pains, the head well down and properly rotated, the perineum was as unyielding as a stone wall. No softening whatever, the head simply forced through the perineal body from force of the pains, the tear extending to, but not involving, the rectal mucous membrane. This case might have had a more fortunate ending in the hands of some of the more experienced of you, yet I doubt it.

My rule, after making one or more examinations and satisfying myself that the os is not dilating rapidly enough, is to administer fifteen grains chloral hydrate at a dose by the mouth, and insert partly within the vaginal orifice a piece of cotton saturated with a four per cent. solution of cocaine, allowing to remain for half an hour and renewing if necessary, though at the expiration of that time there is usually complete relaxation, and rapid dilatation taking place, so that at the proper time the perineum readily allows the presenting part to pass without injury to itself, though as an additional safeguard I usually insert a forefinger in the rectum and

---

*Read before the Brazos Valley Medical Association, November 13, 1901, at Bryan, Texas.

press up the perineal body. In place of the cocaine solution I have used applications of belladonna ointment, but not with the same satisfactory results.

After labor is completed I invariably examine for lacerations, and if extensive enough I immediately repair while the parts are in state of partial anesthesia from the shock of labor, taking as many stitches as may be necessary to secure perfect coaptation, using both before and after the operation douche of warm carbolized water. I also apply loose binder to knees and order nurse to draw the urine off every six hours for one week, at the end of which time the stitches may usually be removed. I prefer and always use catgut sutures, though most authorities advise silver wire. In neither of my three cases did the tear extend into the rectum, but in each of them involving the entire perineal body.

I have operated upon several patients for the relief of partial and complete prolapsus uteri, where the cause was clearly traceable to perineal lacerations involving more or less of the perineal body, and overlooked, or wilfully neglected at the time of labor. And of the many hysterical and neurasthenic, overworked and worn out females applying to us for treatment, a goodly per cent. date the beginning of their ailment back to a laceration of the os uteri or perineum, neglected through the lack of knowledge of the *gramy,* or incompetency of the physician.

You can cure them by a proper operation. Very few instruments and no great amount of skill being required.

